# Aromatase inhibition in the treatment of advanced breast cancer: is there a relationship between potency and clinical efficacy?

**DOI:** 10.1038/sj.bjc.6601731

**Published:** 2004-04-13

**Authors:** R Sainsbury

**Affiliations:** 1 Department of Surgery, Royal Free and University College Medical School, Charles Bell House, 67–73 Riding House Street, London W1W 7EJ, UK

**Keywords:** anastrozole, aromatase inhibition, oestrogen suppression, potency, clinical efficacy

## Abstract

Two-thirds of breast tumours are oestrogen-receptor positive and 60–70% of these tumours respond to interventions that reduce the effects of oestrogen. Until recently, tamoxifen was the drug of choice for the treatment of hormone-responsive early and advanced breast cancer. However, tamoxifen is associated with increased incidences of endometrial cancer and thromboembolic disease, and many tumours eventually become resistant to treatment with tamoxifen. Thus, there is a need for alternative therapies with different mechanisms of action. In postmenopausal women, aromatase inhibitors (AIs) suppress oestrogen levels by inhibiting oestrogen synthesis via the aromatase enzyme pathway. The third-generation AIs (anastrozole, letrozole and exemestane) are more potent than the earlier AIs (aminoglutethimide, formestane and fadrozole) with respect to both aromatase inhibition and oestrogen suppression. While the earlier AIs were unable to show any benefit over megestrol acetate or tamoxifen as second- and first-line therapy, respectively, in postmenopausal women with advanced breast cancer, third-generation AIs have shown significant benefits in both settings. Comparison of aromatase inhibition and oestrogen suppression between the third-generation AIs anastrozole and letrozole showed a small but significantly greater difference in the degree of suppression of oestrone and oestrone sulphate (but not oestradiol), with letrozole. In an open-label trial, there were no significant differences between letrozole and anastrozole for the clinical end points of time to progression (primary end point), time to treatment failure, overall survival, clinical benefit, duration of clinical benefit, time to response, duration of response or objective response rate in patients with confirmed hormone receptor-positive tumours. Together these data suggest that once a certain threshold of aromatase inhibition is reached, small differences in oestrogen suppression between the third-generation AIs do not lead to clinically significant differences in overall efficacy.

Breast cancer is regarded world wide as a major cause of morbidity and mortality in both pre- and postmenopausal women, and currently comprises 18% of all female cancers (McPherson *et al*, 2000). In the UK, breast cancer accounts for more than 14 000 deaths each year (McPherson *et al*, 2000), while in Europe in 1995 it was responsible for the deaths of 124 000 women (Bray *et al*, 2002). However, between 1988 and 1998 mortality from the disease fell dramatically among women below the age of 70 years in Western (but not Eastern) Europe (Levi *et al*, 2001). Possible reasons for this improved mortality rate include the introduction of national breast screening programmes and new better treatment regimens (Blanks *et al*, 2000; Levi *et al*, 2001).

It is recognised that two-thirds of breast tumours are oestrogen-receptor positive and women who have hormone receptor-positive tumours are suitable candidates for endocrine therapy (Forbes, 1997). The use of endocrine therapy for the management of the disease has grown dramatically since the first pioneering report by Beatson in 1896 of a successful outcome in a premenopausal woman with breast cancer following ovarian ablation (Beatson, 1896), and many different treatment approaches are now available. Although significant progress has been made over the past three decades in terms of both improved efficacy and tolerability of endocrine treatments for breast cancer, many challenges still lie ahead for both surgeons and oncologists in the treatment and management of all stages of the disease.

Until recently, and for more than 30 years, tamoxifen – a selective oestrogen receptor modulator (SERM) – had been the drug of choice for the treatment of hormone-responsive early and advanced breast cancer. However, although tamoxifen is an effective treatment, it has partial agonist activity. This is associated with an increase in the incidence of endometrial cancer (Fisher *et al*, 1996) and of thromboembolic disease (Jaiyesimi *et al*, 1995; Fisher *et al*, 1996), and limits its use. In addition, most tumours eventually become resistant to tamoxifen and alternative treatments are required. In recent years, a number of different classes of endocrine therapy have emerged as suitable alternatives in the treatment of advanced breast disease. Although several new SERMs have been developed (e.g. raloxifene), they have not been shown to produce any clinically relevant effects in the treatment of tamoxifen-resistant tumours (Johnston, 2001). Thus, there is a need for new therapies with improved tolerability profiles that are not cross-resistant with established endocrine therapies such as tamoxifen. The availability of endocrine agents with different mechanisms of action to tamoxifen, such as the aromatase inhibitors (AIs), is an important step forward in the search to provide more efficacious and better-tolerated therapies.

The AIs have been developed for the treatment of women with breast cancer in whom ovarian function has ceased either due to the menopause or as a result of ovarian ablation, through oophorectomy or by ovarian irradiation. They prevent the formation of oestrogen from androgens in postmenopausal women through inhibition of the cytochrome *P*450 enzyme, aromatase, which catalyses the conversion of androgens to oestrogens in the fat, liver and muscle cells (Dowsett *et al*, 1995; Geisler *et al*, 1996) and breast tumour tissue itself (Bhatnagar *et al*, 2001; Geisler *et al*, 2001). In premenopausal women, the ovaries are the primary site of oestrogen production and AIs are not able to completely block ovarian oestrogen synthesis.

Aminoglutethimide was the first AI to become available in the late 1970s (Wells *et al*, 1978) and the first to show efficacy as second-line therapy after tamoxifen in postmenopausal women with advanced hormone-responsive breast cancer. However, its toxicity and lack of selectivity for the aromatase enzyme, necessitating concomitant corticosteroid supplementation (Wells *et al*, 1978), prevented it from becoming a more widely used treatment. Formestane, a steroidal AI, became available in 1993. It was also effective for treatment of postmenopausal women with advanced breast cancer, but more selective than aminoglutethimide, and therefore associated with fewer side effects. However, as a result of extensive first-pass metabolism, formestane cannot be given orally and has to be administered twice-monthly by intramuscular injection, leading to reports of local reactions in up to 17% of patients (Goss *et al*, 1986).

The newer third-generation AIs, which include the nonsteroidal agents, anastrozole, letrozole and fadrozole (Japan only) and the steroidal compound, exemestane (Figure 1

**Figure 1:**
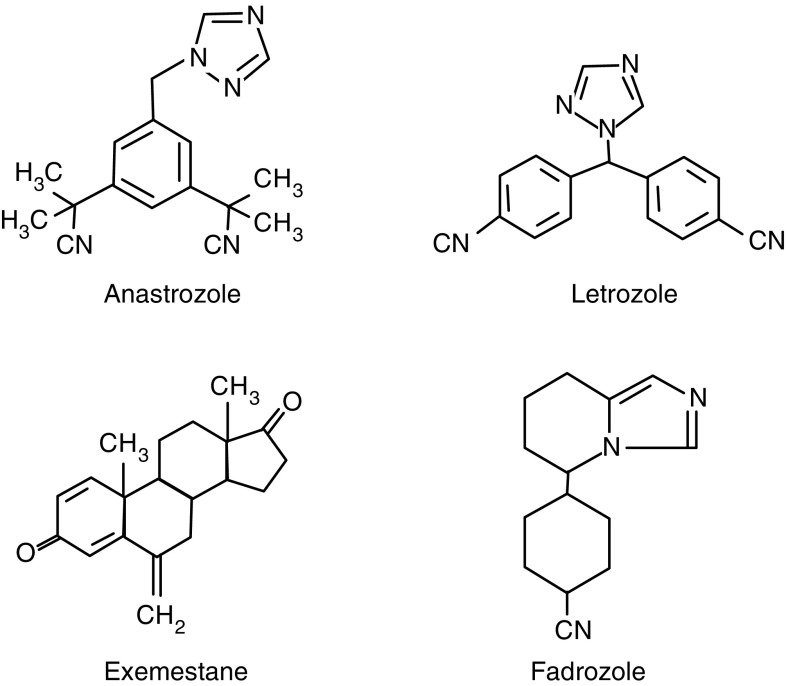
Structures of anastrozole, letrozole, exemestane and fadrozole.

), are the most recent AIs to become available for use in postmenopausal women with metastatic hormone-responsive breast tumours. These AIs show increased potency with respect to both aromatase inhibition and oestrogen suppression compared with the earlier AIs. Small but statistically significant differences in potency have also been reported between third-generation AIs (Geisler *et al*, 2002).

An important question is whether or not these differences in potency between the third-generation AIs lead to clinically relevant differences in efficacy and tolerability. This paper reviews current published data on potency and clinical efficacy to determine if any such relationship exists.

## WHOLE-BODY OESTROGEN SUPPRESSION AND AROMATASE INHIBITION

### Comparison of first- and second- *vs* third-generation aromatase inhibitors: oestrogen suppression and aromatase inhibition

**Figure 2:**
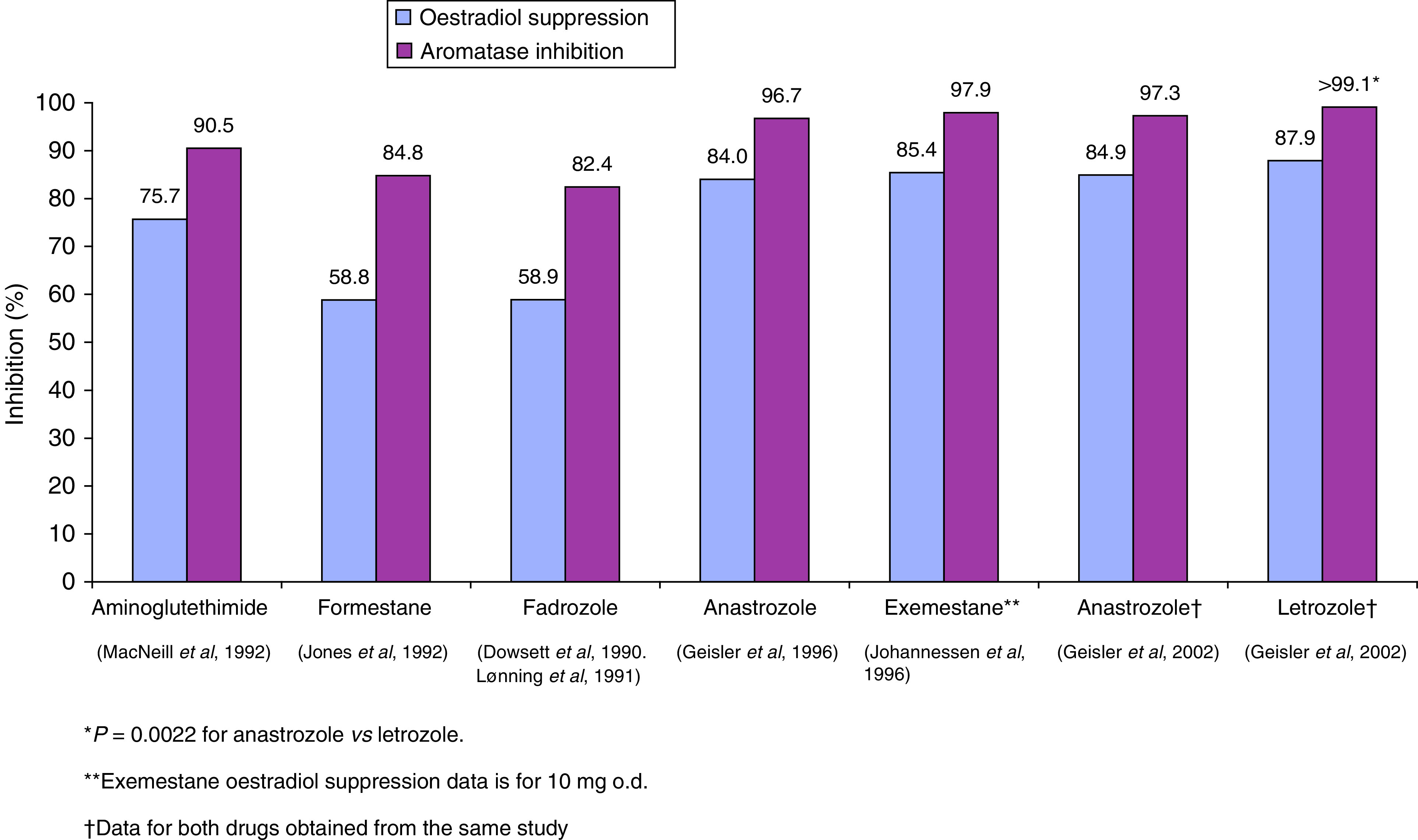
Indirect comparison of oestradiol suppression and aromatase inhibition by first- and second- *vs* third-generation aromatase inhibitors (Dowsett *et al*, 1990; Lønning *et al*, 1991; Jones *et al*, 1992; MacNeill *et al*, 1992; Geisler *et al*, 1996; Johannessen *et al*, 1997; Geisler *et al*, 2002). ^*^P=0.0022 for anastrozole *vs* letrozole. ^**^Exemestane oestradiol suppression data is for 10 mg o.d. ^†^Data for both drugs obtained from the same study.

Indirect comparisons of oestrogen suppression by the first- and second-generation AIs aminoglutethimide, formestane and fadrozole and the third-generation AIs anastrozole and exemestane that were conducted in the same laboratory, have shown that third-generation AIs are more potent than the earlier AIs (Figure 2

). These studies demonstrated that aminoglutethimide (1000 mg once daily (o.d.)) suppressed oestradiol levels by 75% (MacNeill *et al*, 1992), while formestane (250 mg o.d.) and fadrozole (1 mg twice daily) suppressed oestradiol by approximately 59% (Dowsett *et al*, 1990; Lønning *et al*, 1991; Jones *et al*, 1992). Anastrozole (1 mg o.d.) and exemestane (10 mg o.d.) showed similar activities to each other (84 *vs* 85%, respectively) and were more potent than aminoglutethimide, formestane and fadrozole (Geisler *et al*, 1996; Johannessen *et al*, 1997). Similar differences between the agents were observed with respect to aromatase inhibition (Figure 2). An unrelated study comparing anastrozole (1 mg o.d.) with letrozole (2.5 mg o.d.) (Geisler *et al*, 2002) showed that anastrozole was as potent as letrozole in terms of oestradiol suppression (84.9 *vs* 87.8%, respectively, *P*=0.1088), although the difference in aromatase inhibition between anastrozole and letrozole was significant (97.3 *vs* >99.1%, respectively, *P*=0.0022) (Figure 2) (Geisler *et al*, 2002).

### Direct comparison of third-generation aromatase inhibitors, anastrozole and letrozole: oestrogen suppression

**Figure 3:**
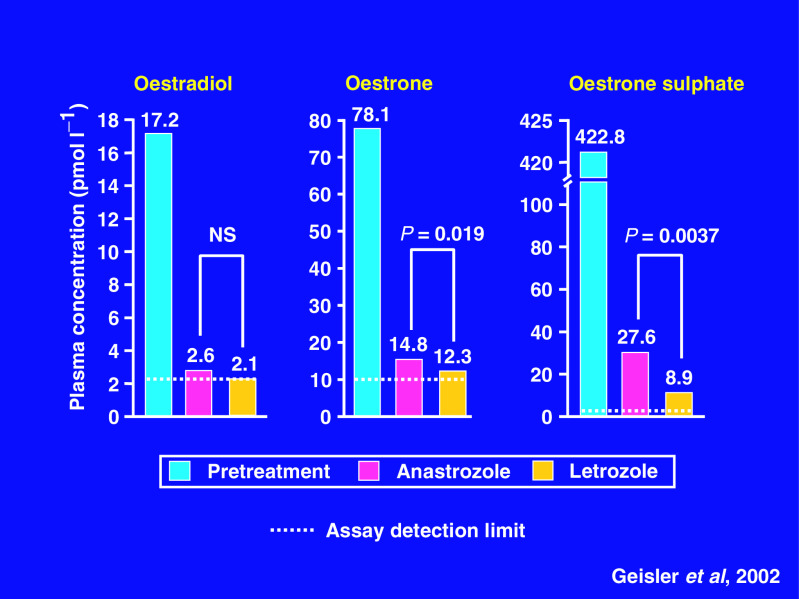
Plasma oestrogen levels in postmenopausal women with advanced breast cancer (Geisler *et al*, 2002).

The study in which anastrozole was directly compared with letrozole (Geisler *et al*, 2002) was a small-scale, randomised, double-blind, cross-over trial. In all, 12 postmenopausal women with oestrogen receptor-positive metastatic tumours suitable for treatment with AIs were enroled into the study. Six patients received 6 weeks of anastrozole (1 mg o.d.) followed by 6 weeks of letrozole (2.5 mg o.d.), while the other six received 6 weeks of letrozole followed by 6 weeks of anastrozole. Compared with anastrozole, letrozole showed small but significantly increased suppression of oestrone and oestradiol sulphate concentrations (Figure 3

), but as discussed above, there was no difference in suppression of oestradiol (Geisler *et al*, 2002).

## CLINICAL EFFICACY

### Comparison of the third-generation aromatase inhibitors with megestrol acetate and aminoglutethimide as second-line therapy for advanced breast cancer

Several Phase III studies have compared the efficacy of the third-generation AIs in postmenopausal women with advanced breast cancer who have progressed on tamoxifen, *vs* the previous standard treatments in this setting, megestrol acetate or aminoglutethimide (Table 1

**Table 1:** Overview of efficacy results from Phase III trials of second-line treatment of advanced breast cancer. Aromatase inhibitors *vs* megestrol acetate and aminoglutethimide in patients who have failed on tamoxifen (Buzdar *et al*, 1996a, b; 1998; Dombernowsky *et al*, 1998; Gershanovich *et al*, 1998; Chaudri and Trunet, 1999; Kaufmann *et al*, 2000; Buzdar *et al*, 2001)

	**European & US combined analysis**		**European trial**			**US trial**			**International trial**			**International trial**	
	**A**	**MA**	**L**		**MA**	**L**		**MA**	**L**		**AG**	**E**	**MA**
Dose	1 mg o.d. (*n*=263)	40 mg q.d. (*n*=253)	0.5 mg o.d. (*n*=188)	2.5 mg o.d. (*n*=174)	40 mg q.d. (*n*=189)	0.5 mg o.d. (*n*=202)	2.5 mg o.d. (*n*=199)	40 mg q.d. (*n*=201)	0.5 mg o.d. (*n*=192)	2.5 mg o.d. (*n*=185)	250 mg b.d. (*n*=178)	2.5 mg o.d. (*n*=366)	40 mg q.d. (*n*=403)
Median follow-up (months)	31		33			37			20			11	
Median TTP (months)	4.8	4.6	5.1	5.6	5.5	6.0	3.0	6.0	3.3	3.4	3.2	4.7	3.8
	ns		ns			0.5 mg *vs* MA, *P*=0.044			2.5 mg *vs* AG, *P*=0.008			*P*=0.037	
Median survival (months)	26.7	22.5	21.5^a^	25.3^a^	21.5^a^	33	29	26	21	28	20	NR	28.4
	*P*<0.025		ns			ns			2.5 mg *vs* AG, *P*=0.002			*P*=0.039	

a Survival data from an extended 51-month follow-up analysis. A=anastrozole; MA=megestrol acetate; L=letrozole; AG=aminoglutethimide; E=exemestane; o.d.=once daily; q.d.=four times daily; b.d.=twice daily; TTP=time to disease progression; NR=not reached; ns=nonsignificant.

).

The efficacy of anastrozole (1 mg or 10 mg od) compared with megestrol acetate [40 mg four times daily (qd)] has been assessed in two trials, one European (Jonat *et al*, 1996) and one North American (Buzdar *et al*, 1997), prospectively planned for combined analysis (Buzdar *et al*, 1996a). The results summarized in Table 1 are of the combined analysis.

After a median follow-up of 6 months, time to progression (TTP) and objective response (OR=complete+partial response) rate did not differ significantly for the 1 and 10 mg anastrozole groups compared with the megestrol acetate group (Table 1). The overall median TTP was approximately 21 weeks and approximately one-third of patients in each treatment group benefited from therapy (Buzdar *et al*, 1996a). After 31 months of follow-up (Buzdar *et al*, 1998), anastrozole (1 mg) demonstrated a significant survival advantage over megestrol acetate (Table 1). There was no significant difference for overall survival between anastrozole 10 mg and megestrol acetate, although numerical advantages have been shown in favour of anastrozole 10 mg, with longer median time to death (25.5 *vs* 22.5 months for anastrozole 10 mg and megestrol acetate, respectively; *P*=0.09) and lower death rate at 2 years (45.4 *vs* 53.7%, respectively). Therefore, while there was no advantage for the higher dose of anastrozole, the data are consistent with, and supportive, of the findings observed with the clinically approved 1 mg dose.

Two trials have investigated the efficacy of letrozole (2.5 or 0.5 mg o.d.) *vs* megestrol acetate 40 mg q.d. (Dombernowsky *et al*, 1998; Chaudri and Trunet, 1999; Buzdar *et al*, 2001) (Table 1). Results of the European trial (Dombernowsky *et al*, 1998) showed the OR rate was significantly higher for patients receiving letrozole 2.5 mg than for letrozole 0.5 mg (*P*=0.004) or megestrol acetate (*P*=0.04) (24, 13 and 16%, respectively). Letrozole 2.5 mg was superior to letrozole 0.5 mg for TTP (*P*=0.02), but not compared with megestrol acetate. At a 51-month follow-up analysis for the letrozole 2.5 mg arm (Chaudri and Trunet, 1999), letrozole did not show significant survival benefit over megestrol acetate (Table 1). In the US trial (Buzdar *et al*, 2001), no significant differences were reported for TTP (Table 1) or OR rate and, contrary to the first trial, the efficacy of letrozole was not dose related. No significant survival benefit was seen for letrozole *vs* megestrol acetate (Table 1). It has been speculated that these contradictory findings may be the result of an imbalance of prognostic factors in favour of megestrol acetate in the latter trial (Wischnewsky *et al*, 2002).

In a further open-label trial, letrozole (2.5 and 0.5 mg o.d.) was compared with aminoglutethimide (250 mg, twice daily) (Gershanovich *et al*, 1998) (Table 1). The higher dose of letrozole (2.5 mg daily) was superior to aminoglutethimide for overall survival (*P*=0.002), TTP (Cox regression analysis, *P*=0.008) and TTF (Cox regression analysis, *P*=0.003). There were no significant differences in OR seen in patients receiving letrozole 2.5 mg, letrozole 0.5 mg or aminoglutethimide. Letrozole 2.5 mg showed a significant advantage over the letrozole 0.5 mg dose for survival (*P*=0.04), but there was not a significant dose–response effect for letrozole in terms of TTP, in line with the results from the US trial of letrozole *vs* megestrol acetate (Buzdar *et al*, 2001). In addition, a trial that assessed the impact of letrozole 2.5 and 0.5 mg o.d. on peripheral aromatisation of androstenedione to oestrone has shown no differences between doses in inhibition of aromatisation (Dowsett *et al*, 1995). All patients on the lower dose showed >97% inhibition and on the higher dose showed >98% inhibition. There was no evidence of any difference between letrozole 2.5 and 0.5 mg in suppression of oestrone (80.8 and 82.0%, respectively) and oestradiol (68.1 and 84.1%, respectively), but no formal statistical analysis was performed. These results draw into question the dose–response effect seen for letrozole in the European trial of letrozole *vs* megestrol acetate (Dombernowsky *et al*, 1998; Chaudri and Trunet, 1999).

A large, randomised, double-blind trial has compared exemestane (25 mg o.d.) with megestrol acetate (40 mg q.d.) (Kaufmann *et al*, 2000). There was significant improvement in TTP for exemestane compared with megestrol acetate (Table 1), while OR rate was not significantly different between groups. Exemestane also showed significant improvement in overall survival compared with megestrol acetate (Table 1), although this was after a shorter median follow-up (11.4 months) compared with the anastrozole and letrozole studies.

Overall, although there have been some differences in outcome and the end points at which significant benefits have been observed, the newer-generation AIs have all proven more effective and better tolerated than the progestogen megestrol acetate, and letrozole has demonstrated superior efficacy to aminoglutethimide, for the second-line treatment of patients with advanced breast cancer failing on tamoxifen. As a result, third-generation AIs are now established as the standard treatment in this patient population. In contrast, the second-generation AIs formestane and fadrozole have shown no significant efficacy benefits over megestrol acetate in patients progressing on tamoxifen (Buzdar *et al*, 1996b; Thürlimann *et al*, 1997).

### Comparison of the third-generation aromatase inhibitors with tamoxifen as first-line therapy for advanced breast cancer

Results of the Phase III studies assessing the efficacy of the third-generation AIs *vs* tamoxifen as first-line therapy in postmenopausal women with advanced breast cancer are summarised in Table 2

**Table 2:** Nonsteroidal aromatase inhibitors *vs* tamoxifen as first-line treatment

	**TARGET study (Bonneterre *et al*, 2000)**		**N American study (Nabholtz *et al*, 2000)**		**Combined study (Bonneterre *et al*, 2000)**		**Letrozole study (Mouridsen *et al*, 2001)**	
	**A**	**T**	**A**	**T**	**A**	**T**	**L**	**T**
	***n*=340**	***n*=328**	***n*=171**	***n*=182**	***n*=511**	***n*=510**	***n*=453**	***n*=454**
TTP overall population (months)	8.2	8.3	11.1	5.6	8.5	7.0	9.4	6.0
	NS		*P*=0.005		NS		*P*=0.0001	
TTP HR+ve subgroup (months)	8.9	7.8	NA		10.7	6.4	9.2	5.8
	NA				*P*=0.022		*P*=0.0001	
CB (%)	56.2	55.5	59.1	45.6	57.1	52.0	49.0	38.0
	NA		*P*=0.0098		*P*=0.1129		*P*=0.001	
OR (%)	32.9	32.9	21.0	17.0	29.0	27.1	30.0	20.0
	NS		NA		NS		*P*=0.0006	
HR+ve (%)	45		89		60		66	

A=anastrozole; T=tamoxifen; L=letrozole; TTP=time to disease progression; NA=not available; HR+ve=hormone receptor-positive; CB=clinical benefit; OR=objective response; NS=not significant.

. These data indicate the superiority of the third-generation nonsteroidal AIs compared with tamoxifen in this patient population.

The efficacy of anastrozole *vs* tamoxifen was assessed in two Phase III trials, one European (the Tamoxifen and Arimidex Randomized Group Efficacy and Tolerability (TARGET) trial) (Bonneterre *et al*, 2000) and one North American (Nabholtz *et al*, 2000), which were identical in design and prospectively planned for combined analysis. Anastrozole was shown to be at least as effective as tamoxifen as first-line treatment of postmenopausal women with advanced breast cancer, although some variation in data was observed between the two individual Phase III trials (depending upon the proportion of patients whose tumours were hormone receptor-positive). In the North American trial, TTP and clinical benefit (CB) rates were significantly better for anastrozole compared with tamoxifen (Table 2). In the TARGET trial, anastrozole was shown to be equivalent to tamoxifen in terms of TTP and OR rates (Table 2). The difference in outcome was attributed to differences in the proportion of patients with confirmed hormone receptor-positive tumours (89 *vs* 45% for the North American and TARGET trials, respectively). Analysis of the subgroup that comprised only patients with hormone receptor-positive tumours (45%) in the TARGET trial showed a similar separation of the Kaplan–Meier curves to that seen in the overall population in the North American trial (in which 89% of patients had hormone receptor-positive tumours). The combined analysis of the two trials also showed anastrozole to be superior to tamoxifen for TTP in patients with hormone-sensitive advanced breast cancer (Table 2) (Bonneterre *et al*, 2001).

In a single Phase III study in postmenopausal women with advanced breast cancer, letrozole was also found to be superior to tamoxifen for several efficacy end points (Mouridsen *et al*, 2001). Time to progression, CB and OR rates were significantly better for letrozole (Table 2). In addition, TTF was significantly longer for letrozole compared with tamoxifen (median TTF: 9.2 *vs* 5.7 months, respectively, *P*=0.0001).

Although no data from Phase III trials of exemestane are currently available, the results of a Phase II trial of exemestane (25 mg o.d., *n*=31) *vs* tamoxifen (20 mg o.d., *n*=32) are promising (Paridaens *et al*, 2000). Median TTP was 8.9 *vs* 5.2 months for exemestane and tamoxifen, respectively, and OR rates were 42 *vs* 16%, respectively. A Phase III trial is ongoing.

In contrast to the third-generation AIs, the second-generation agents fadrozole and formestane have not shown any significant efficacy benefits over tamoxifen in the advanced disease setting (Perez Carrion *et al*, 1994; Falkson and Falkson, 1996).

### Direct comparison of the third-generation aromatase inhibitors: anastrozole *vs* letrozole as second-line therapy

Anastrozole (1 mg o.d.) and letrozole (2.5 mg o.d.) were compared as second-line treatment for advanced breast cancer with hormone receptor-positive or unknown receptor status in postmenopausal women who had progressed on tamoxifen in a multicentre, open-label, randomised Phase III–IV study (Rose *et al*, 2002). The primary end point was TTP and secondary end points included OR rate, response duration, duration of clinical benefit, TTF, time to response and overall survival (OS).

A total of 713 postmenopausal patients were randomly allocated to either letrozole 2.5 mg o.d. (*n*=356) or anastrozole 1 mg o.d. (*n*=357). Patient characteristics were well balanced between treatment groups (Rose *et al*, 2002). A total of 48% of patients had hormone receptor-positive tumours (Rose *et al*, 2002). Efficacy end points are shown in Table 3

**Table 3:** Efficacy data in patients randomised to anastrozole or letrozole (Rose *et al*, 2002)

	**ITT population**		
	**Anastrozole (*n*=357)**	**Letrozole (*n*=356)**	***P-*value**
Median TTP^a^ (months)	5.7	5.7	0.920
Median TTF (months)	5.6	5.6	0.761
Median OS (months)	20.3	22.0	0.624
			
Objective response (%)			
Total population	12.3	19.1	0.014
HR+ve subgroup	16.8	17.3	NA
Unknown receptor status subgroup	8.4	20.8	NA

a TTP: primary end point; ITT=intention to treat; TTP=time to disease progression; TTF=time to treatment failure; OS=overall survival.

. In the overall population, anastrozole was similar to letrozole for TTP (*P*=0.920), TTF (*P*=0.761) and OS (*P*=0.624). The only differences between anastrozole and letrozole were for OR rate in both the overall population and the unknown receptor status subgroup, which were higher in the letrozole *vs* the anastrozole group (overall population: 19.1 *vs* 12.3%, odds ratio=1.70, *P*=0.014; unknown receptor status subgroup: 20.8 *vs* 8.4%, respectively) (Rose *et al*, 2002). However, in patients with confirmed hormone receptor-positive tumours there was no difference in OR between anastrozole and letrozole (28/167 [16.8%] *vs* 30/173 [17.3%], respectively). As the overall population included patients with unknown receptor status, it is possible that in this unknown receptor group there was a greater number of patients randomised to letrozole who had hormone receptor-positive tumours and who would respond to letrozole. If this was the case, it could account for the higher OR rate with letrozole in the overall population. Presently, the number of patients with unknown receptor status in each group has not been published. Furthermore, no results for TTP in the hormone receptor-positive population have been published to date, so the relative efficacy of anastrozole and letrozole for this end point is uncertain.

## SUMMARY

The AIs have been developed for treatment of breast cancer in postmenopausal women with hormone receptor-positive tumours and it is important that they are used to treat this group of patients. Third-generation AIs (anastrozole, letrozole and exemestane) show improved potency with respect to suppression of aromatase activity and circulating oestrogen levels compared with the older-generation AIs (aminoglutethimide, formestane and fadrozole) (Dowsett *et al*, 1990; Lønning *et al*, 1991; Jones *et al*, 1992; MacNeill *et al*, 1992; Geisler *et al*, 1996). This increased potency correlates with improved clinical efficacy of the third-generation AIs relative to the older drugs. Thus, while formestane and fadrozole, which inhibit aromatase by <85%, have shown no benefits over megestrol acetate (Buzdar *et al*, 1996b; Thürlimann *et al*, 1997) or tamoxifen (Perez Carrion *et al*, 1994; Falkson and Falkson, 1996) as second- and first-line therapy, respectively, third-generation AIs, which inhibit aromatase activity by >96%, show significant clinical efficacy benefits over these standard second- and first-line comparators (Buzdar *et al*, 1996a, 1998, 2001; Dombernowsky *et al*, 1998; Bonneterre *et al*, 2000; Kaufmann *et al*, 2000; Nabholtz *et al*, 2000; Bonneterre *et al*, 2001; Mouridsen *et al*, 2001).

In contrast, direct comparison of the third-generation AIs anastrozole and letrozole has shown that although letrozole suppresses aromatase activity, oestrone and oestrone sulphate levels to a greater degree than anastrozole, these differences in potency do not translate to clinically significant differences in the efficacy of these agents for the second-line treatment of hormone-sensitive advanced breast cancer (Rose *et al*, 2002).

The fact that these marginal differences in potency between anastrozole and letrozole do not appear to produce clinically relevant differences in efficacy suggests that there may be a threshold effect for aromatase inhibition/oestrogen suppression beyond which no further improvements in clinical efficacy can be gained. Since the potency of formestane, aminoglutethimide and fadrozole fall below this threshold, the third-generation AIs show clinical efficacy benefits relative to these older agents. Of note, however, is that although clinical efficacy may be unaffected by small differences in potency, this does not preclude the fact that small differences in oestrogen suppression may lead to differences in side-effect profiles, especially with respect to cardiovascular end points or effects on bone mineral density, and this is more likely to become apparent during longer-term treatment as these drugs move into the adjuvant setting.

## CONCLUSION

The third-generation AIs are more potent inhibitors of the aromatase enzyme and cause greater oestrogen suppression than older agents such as aminoglutethimide, fadrozole and formestane. This is linked to an increase in the clinical efficacy of the third-generation AIs relative to the previous standard comparators in both the second- and first-line settings. In contrast, small changes in potency between anastrozole and letrozole are not associated with clinically relevant differences in their efficacy in the treatment of hormone-sensitive advanced breast cancer, suggesting the existence of a threshold of oestrogen suppression above which no further improvements in clinical efficacy can be gained.

## References

[bib1] Beatson GT (1896) On the treatment of inoperable cases of carcinoma of the mamma; suggestions for a new method of treatment with illustrative cases. Lancet 2: 104–107 PMC5518378 29584099

[bib2] Bhatnagar AS, Brodie AMH, Long BJ, Evans DB, Miller WR (2001) Intracellular aromatase and its relevance to the pharmacological efficacy of aromatase inhibitors. J Steroid Biochem Mol Biol 76: 199–202 11384878 10.1016/s0960-0760(01)00050-4

[bib3] Blanks RG, Moss SM, McGahan CE, Quinn MJ, Babb PJ (2000) Effect of NHS breast screening programme on mortality from breast cancer in England and Wales, 1990–8: comparison of observed with predicted mortality. BMJ 321: 665–669 10987769 10.1136/bmj.321.7262.665 PMC27479

[bib4] Bonneterre J, Buzdar A, Nabholtz JM, Robertson JF, Thurlimann B, von Euler M, Sahmoud T, Webster A, Steinberg M, Arimidex Writing Committee; Investigators Committee Members (2001) Anastrozole is superior to tamoxifen as first-line therapy in hormone receptor positive advanced breast carcinoma. Cancer 92: 2247–2258 11745278 10.1002/1097-0142(20011101)92:9<2247::aid-cncr1570>3.0.co;2-y

[bib5] Bonneterre J, Thürlimann BJK, Robertson JFR (2000) Anastrozole *versus* tamoxifen as first-line therapy for advanced breast cancer in 668 postmenopausal women: results of the tamoxifen or Arimidex randomized group efficacy and tolerability study. J Clin Oncol 18: 3748–3757 11078487 10.1200/JCO.2000.18.22.3748

[bib6] Bray F, Sankila R, Ferlay J, Parkin DM (2002) Estimates of cancer incidence and mortality in Europe in 1995. Eur J Cancer 38: 99–166 11750846 10.1016/s0959-8049(01)00350-1

[bib7] Buzdar A, Douma J, Davidson N, Elledge R, Morgan M, Smith R, Porter L, Nabholtz J, Xiang X, Brady C (2001) Phase III, multicenter, double-blind, randomized study of letrozole, an aromatase inhibitor, for advanced breast cancer *versus* megestrol acetate. J Clin Oncol 19: 3357–3366 11454883 10.1200/JCO.2001.19.14.3357

[bib8] Buzdar A, Jonat W, Howell A, Jones SE, Blomqvist C, Vogel CL, Eiermann W, Wolter JM, Azab M, Webster A, Plourde PV (1996a) Anastrozole, a potent and selective aromatase inhibitor, *versus* megestrol acetate in postmenopausal women with advanced breast cancer: results of overview analysis of two Phase III trials. J Clin Oncol 14: 2000–2011 8683230 10.1200/JCO.1996.14.7.2000

[bib9] Buzdar AU, Jonat W, Howell A, Jones SE, Blomqvist CP, Vogel CL, Eiermann W, Wolter JM, Steinberg M, Webster A, Lee D (1998) Anastrozole *versus* megestrol acetate in the treatment of postmenopausal women with advanced breast carcinoma. Results of a survival update based on a combined analysis of data from two mature Phase III trials. Cancer 83: 1142–1152 9740079

[bib10] Buzdar AU, Smith R, Vogel C, Bonomi P, Keller AM, Favis G, Mulagha M, Cooper J (1996b) Fadrozole HCl (CGS-16949A) *versus* megestrol acetate treatment of postmenopausal patients with metastatic breast carcinoma. Cancer 77: 2503–2513 8640699 10.1002/(SICI)1097-0142(19960615)77:12<2503::AID-CNCR13>3.0.CO;2-W

[bib11] Buzdar AU, Smith R, Vogel C, Bonomi P, Keller AM, Favis G, Mulagha M, Cooper J (1997) A Phase III trial comparing anastrozole (1 and 10 mg), a potent and selective aromatase inhibitor, with megestrol acetate in postmenopausal women with advanced breast cancer. Cancer 79: 730–739 9024711

[bib12] Chaudri HA, Trunet PF (1999) Letrozole. Updated duration of response. J Clin Oncol 17: 3859–3860 10.1200/JCO.1999.17.12.3856 10577865

[bib13] Dombernowsky P, Smith I, Falkson G, Leonard R, Panasci L, Bellmunt J, Bezwoda W, Gardin G, Gudgeon A, Morgan M, Fornasiero A, Hoffmann W, Michel J, Hatschek T, Tjabbes T, Chaudri HA, Hornberger U, Trunet PF (1998) Letrozole, a new oral aromatase inhibitor for advanced breast cancer: double-blind randomized trial showing a dose effect and improved efficacy and tolerability compared with megestrol acetate. J Clin Oncol 16: 453–461 9469328 10.1200/JCO.1998.16.2.453

[bib14] Dowsett M, Jones A, Johnston SR, Jacobs S, Trunet P, Smith IE (1995) *In vivo* measurements of aromatase inhibition by letrozole (CGS 20267) in postmenopausal patients with breast cancer. Clin Cancer Res 1: 1511–1515 9815951

[bib15] Dowsett M, Stein RC, Mehta A, Coombes RC (1990) Potency and selectivity of the non-steroidal aromatase inhibitor CGS 16949A in postmenopausal breast cancer patients. Clin Endocrinol 32: 623–634 10.1111/j.1365-2265.1990.tb00906.x 2142026

[bib16] Falkson CI, Falkson HC (1996) A randomised study of CGS 16949A (fadrozole) *versus* tamoxifen in previously untreated postmenopausal patients with metastatic breast cancer. Ann Oncol 7: 465–469 8839900 10.1093/oxfordjournals.annonc.a010634

[bib17] Fisher B, Dignam J, Bryant J, Wolmark N (1996) Five *versus* more than five years of tamoxifen therapy for breast cancer patients with negative lymph nodes and estrogen receptor-positive tumors. J Natl Cancer Inst 88: 1529–1542 8901851 10.1093/jnci/88.21.1529

[bib18] Forbes JF (1997) The control of breast cancer: the role of tamoxifen. Semin Oncol 24(Suppl 1): S1–S5 S1–S19 9045316

[bib19] Geisler J, Detre S, Berntsen H, Ottestad L, Lindtjorn B, Dowsett M, Einstein Lonning P (2001) Influence of neoadjuvant anastrozole (Arimidex) on intratumoral estrogen levels and proliferation markers in patient with locally advanced breast cancer. Clin Cancer Res 7: 1230–1236 11350888

[bib20] Geisler J, Haynes B, Anker G, Dowsett M, Lønning PE (2002) Influence of letrozole and anastrozole on plasma estrogen levels in postmenopausal breast cancer patients evaluated in a randomized, cross-over study. J Clin Oncol 20: 751–757 11821457 10.1200/JCO.2002.20.3.751

[bib21] Geisler J, King N, Dowsett M, Ottestad L, Lundgren S, Walton P, Kormeset PO, Lonning PE (1996) Influence of anastrozole (Arimidex), a selective, non-steroidal aromatase inhibitor, on *in vivo* aromatisation and plasma oestrogen levels in postmenopausal women with breast cancer. Br J Cancer 74: 1286–1291 8883419 10.1038/bjc.1996.531 PMC2075919

[bib22] Gershanovich M, Chaudri HA, Campos D, Lurie H, Bonaventura A, Jeffrey M, Buzzi F, Bodrogi I, Ludwig H, Reichardt P, O'Higgins N, Romieu G, Friederich P, Lassus M (1998) Letrozole, a new oral aromatase inhibitor: randomized trial comparing 2.5 mg daily, 0.5 mg daily and aminoglutethimide in postmenopausal women with advanced breast cancer. Ann Oncol 9: 639–645 9681078 10.1023/a:1008226721932

[bib23] Goss PE, Powles TJ, Dowsett M, Hutchison G, Brodie AMH, Gazet JC, Coombes RC (1986) Treatment of advanced postmenopausal breast cancer with the aromatase inhibitor, 4-hydroxyandrostenedione: Phase II report. Cancer Res 46: 4823–4826 2942241

[bib24] Jaiyesimi IA, Buzdar AU, Decker DA, Hortobagyi GN (1995) Use of tamoxifen for breast cancer: twenty-eight years later. J Clin Oncol 13: 513–529 7844613 10.1200/JCO.1995.13.2.513

[bib25] Johannessen DC, Engan T, Di Salle E, Zurlo MG, Paolini J, Ornati G, Piscitelli G, Kvinnsland S, Lonning PE (1997) Endocrine and clinical effects of exemestane (PNU 155971), a novel steroidal aromatase inhibitor, in postmenopausal breast cancer patients: a Phase I study. Clin Cancer Res 3: 1101–1108 9815789

[bib26] Johnston SRD (2001) Endocrine manipulation in advanced breast cancer: recent advances with SERM therapies. Clin Cancer Res 7: 4376s–4387s 11916228

[bib27] Jonat W, Howell A, Blomqvist C, Eiermann W, Winblad G, Tyrrell C, Mauriac L, Roche H, Lundgren S, Hellmund R, Azab M (1996) A randomised trial comparing two doses of the new selective aromatase inhibitor (‘Arimidex’) with megestrol acetate in postmenopausal women with advanced breast cancer. Eur J Cancer 32A: 404–412 8814682 10.1016/0959-8049(95)00014-3

[bib28] Jones AL, MacNeill F, Jacobs S, Lonning PE, Dowsett M, Powles TJ (1992) The influence of intramuscular 4-hydroxyandrostenedione on peripheral aromatisation in breast cancer patients. Eur J Cancer 28A: 1712–1716 1389491 10.1016/0959-8049(92)90074-c

[bib29] Kaufmann M, Bajetta E, Dirix LY, Fein LE, Jones SE, Zilembo N, Dugardyn JL, Nasurdi C, Mennel RG, Cervek J, Fowst C, Polli A, di Salle E, Arkhipov A, Piscitelli G, Miller LL, Massimini G (2000) Exemestane is superior to megestrol acetate after tamoxifen failure in postmenopausal women with advanced breast cancer: results of a Phase III randomized double-blind study. J Clin Oncol 18: 1399–1411 10735887 10.1200/JCO.2000.18.7.1399

[bib30] Levi F, Lucchini F, Negri E, La Vecchia C (2001) The fall in breast cancer mortality in Europe. Eur J Cancer 37: 1409–1412 11435073 10.1016/s0959-8049(01)00144-7

[bib31] Lønning PE, Jacobs S, Jones A, Haynes B, Powles T, Dowsett M (1991) The influence of CGS 16949A on peripheral aromatisation in breast cancer patients. Br J Cancer 63: 789–793 1828173 10.1038/bjc.1991.175 PMC1972391

[bib32] MacNeill FA, Jones AL, Jacobs S, Lonning PE, Powles TJ, Dowsett M (1992) The influence of aminoglutethimide and its analogue rogletimide on peripheral aromatisation in breast cancer. Br J Cancer 66: 692–697 1419608 10.1038/bjc.1992.339 PMC1977412

[bib33] McPherson K, Steel CM, Dixon JM (2000) ABC of breast diseases. Breast cancer – epidemiology, risk factors and genetics. BMJ 321: 624–628 10977847 10.1136/bmj.321.7261.624 PMC1118507

[bib34] Mouridsen H, Gershanovich M, Sun Y, Perez-Carrion R, Boni C, Monnier A, Apffelstaedt J, Smith R, Sleeboom HP, Janicke F, Pluzanska A, Dank M, Becquart D, Bapsy PP, Salminen E, Snyder R, Lassus M, Verbeek JA, Staffler B, Chaudri-Ross HA, Dugan M (2001) Superior efficacy of letrozole *versus* tamoxifen as first-line therapy for postmenopausal women with advanced breast cancer: results of a Phase III Study of the International Letrozole Breast Cancer Group. J Clin Oncol 19: 2596–2606 11352951 10.1200/JCO.2001.19.10.2596

[bib35] Nabholtz JM, Buzdar A, Pollak M, Harwin W, Burton G, Mangalik A, Steinberg M, Webster A, von Euler M (2000) Anastrozole is superior to tamoxifen as first-line therapy for advanced breast cancer in postmenopausal women: results of a North American multicenter randomized trial. J Clin Oncol 18: 3758–3767 11078488 10.1200/JCO.2000.18.22.3758

[bib36] Paridaens R, Dirix LY, Beex L, Nooij M, Cufer T, Lohrisch C, Biganzoli L, Van Hoorebeeck I, Duchateau L, Lobelle JP, Piccart M (2000) Exemestane (aromasin) is active and well tolerated as first-line hormonal therapy (HT) of metastatic breast cancer (MBC) patients (pts): results of a randomized phase II trial. J Clin Oncol 19: 83a (abs. 316)

[bib37] Perez Carrion R, Alberola Candel V, Calabresi F, Michel RT, Santos R, Delozier T, Goss P, Mauriac L, Feuilhade F, Freue M, Pannuti F, van Belle S, Martinez J, Wehrle E, Royce CM (1994) Comparison of the selective aromatase inhibitor formestane with tamoxifen as first-line hormonal therapy in postmenopausal women with advanced breast cancer. Ann Oncol 5(Suppl 7): S19–S24 7873457

[bib38] Rose C, Vtoraya O, Pluzanska A, Neave F, Clemens M, Chaudri-Ross HA, Wyld P, Lang R (2002) Letrozole (Femara) *vs* anastrozole (Arimidex): second-line treatment in postmenopausal women with advanced breast cancer. Proc Am Soc Clin Oncol, Abstract 131 http://www.asco.org/ac/1,1003,_12-002324-00_29-00A-00_18-002002-00_19-00131,00.asp

[bib39] Thürlimann B, Castiglione M, Hsu-Schmitz SF, Cavalli F, Bonnefoi H, Fey MF, Morant R, Lohnert T, Goldhirsch A (1997) Formestane *versus* megestrol acetate in postmenopausal breast cancer patients after failure of tamoxifen: a Phase III prospective randomized cross over trial of second-line hormonal treatment (SAKK 20/90). Eur J Cancer 33: 1017–1024 9376181 10.1016/s0959-8049(97)00105-6

[bib40] Wells SA, Santen RT, Lipton A, Haagensen Jr DE, Ruby EJ, Harvey H, Dilley WG (1978) Medical adrenalectomy with aminoglutethimide clinical studies in postmenopausal patients with metatastic breast carcinoma. Ann Surg 187: 475–484 646874 10.1097/00000658-197805000-00004 PMC1396547

[bib41] Wischnewsky MB, Schmid P, Possinger K (2002) An analysis of two contradictory, clinical trials of letrozole *versus* megestrol acetate, for the treatment of advanced breast cancer. Breast Cancer Res Treat 76(Suppl 1): S76 abstract 266

